# MAP17 predicts sensitivity to platinum-based therapy, EGFR inhibitors and the proteasome inhibitor bortezomib in lung adenocarcinoma

**DOI:** 10.1186/s13046-018-0871-7

**Published:** 2018-08-17

**Authors:** Irene Ferrer, Álvaro Quintanal-Villalonga, Sonia Molina-Pinelo, Jose Manuel Garcia-Heredia, Marco Perez, Rocío Suárez, Santiago Ponce-Aix, Luis Paz-Ares, Amancio Carnero

**Affiliations:** 1 H12O-CNIO Lung Cancer Clinical Research Unit, Institute i+12O and CNIO, Madrid, Spain; 2 CIBER de Cáncer, ISCIII, Madrid, Spain; 3 Instituto de Biomedicina de Sevilla (IBIS) (HUVR, CSIC, Hospital Universitario Virgen del Rocio, University of Seville, Avda. Manuel Siurot s/n, 41013), Seville, Spain; 4 Department of Vegetal Biochemistry and Molecular Biology, University of Seville, Seville, Spain; 5 Medical Oncology Department, Hospital Universitario Doce de Octubre, Madrid, Spain; 6 University Complutense of Madrid, Madrid, Spain; 7 Present address: Program in Molecular Pharmacology, Memorial Sloan Kettering Cancer Center, New York, NY USA

**Keywords:** Biomarkers, Lung cancer, PDZK1IP1, Treatment efficacy

## Abstract

**Background:**

The high incidence and mortality of lung tumours is a major health problem. Therefore, the identification both of biomarkers predicting efficacy for therapies in use and of novel efficacious therapeutic agents is crucial to increase patient survival. MAP17 (PDZK1IP1) is a small membrane-bound protein whose upregulation is reported as a common feature in tumours from diverse histological origins. Furthermore, MAP17 is correlated with tumour progression.

**Methods:**

We assessed the expression of MAP17 in preclinical models, including cell lines and patient-derived xenografts (PDXs), assessing its correlation with sensitivity to different standard-of-care drugs in lung adenocarcinoma, as well as novel drugs. At the clinical level, we subsequently correlated MAP17 expression in human tumours with patient response to these therapies.

**Results:**

We show that MAP17 expression is induced during lung tumourigenesis, particularly in lung adenocarcinomas, and provide in vitro and in vivo evidence that MAP17 levels predict sensitivity to therapies currently under clinical use in adenocarcinoma tumours, including cisplatin, carboplatin and EGFR inhibitors. In addition, we show that MAP17 expression predicts proteasome inhibitor efficacy in this context and that bortezomib, an FDA-approved drug, may be a novel therapeutic approach for MAP17-overexpressing lung adenocarcinomas.

**Conclusions:**

Our results indicate a potential prognostic role for MAP17 in lung tumours, with particular relevance in lung adenocarcinomas, and highlight the predictive pot0065ntial of this membrane-associated protein for platinum-based therapy and EGFR inhibitor efficacy. Furthermore, we propose bortezomib treatment as a novel and efficacious therapy for lung adenocarcinomas exhibiting high MAP17 expression.

**Electronic supplementary material:**

The online version of this article (10.1186/s13046-018-0871-7) contains supplementary material, which is available to authorized users.

## Background

Pulmonary tumours are a major health problem due to both their high incidence and mortality and are responsible for the majority of cancer-related deaths [1]. Lung cancer is a heterogeneous entity that is classified into two major histologically distinct groups: small cell lung cancer (SCLC) and non-small cell lung cancer (NSCLC). Within NSCLC, different histological subtypes can be further distinguished: large cell carcinoma (LCC), squamous cell carcinoma (SCC), and adenocarcinoma (ADC). ADC is the most prevalent subtype, comprising approximately 50% of all lung cancer cases [2]. The overwhelmingly poor prognosis of patients with lung cancer, with an expected 5-year survival rate of only 18%, is a consequence of both late diagnosis and the relative lack of effective systemic treatments [3]. Relatively recent clinical implementation of a limited number of targeted therapies, such as EGFR and ALK inhibitors, has shown benefit in select patients [4], and recently approved immunotherapies have shown very promising results [5]. However, the gold-standard therapy for most lung cancer patients remains classical chemotherapy, with special emphasis on platinum-based compounds. Thus, the identification of biomarkers predicting efficacy for therapies in use is crucial to improve patient survival in this setting. Furthermore, new therapies are necessary to cover the spectrum of patients for whom currently available treatments are insufficient.

MAP17 (DD96, PDZKIP1) is a small, nonglycosylated membrane-associated protein of 17 kDa located in the plasma membrane and the Golgi apparatus [6], which exerts pro-tumourigenic effects in tumour cells by increasing levels of reactive oxygen species (ROS) [7, 8]. MAP17 overexpression has been observed in a wide variety of human carcinomas [9], including cervical, breast, prostate, and ovarian tumours, where expression of this gene is strongly correlated with tumour progression [9, 10]. Elevated MAP17 levels have been associated with good response to platinum-based compounds in cervical and laryngeal carcinomas [10, 11] and with increased sensitivity to bortezomib (Velcade, PS-341), a proteasome inhibitor approved for the treatment of multiple myeloma and mantle cell lymphoma [12, 13] as well as breast tumours and sarcomas [7, 14, 15]. Therefore, we hypothesize that MAP17 may prove useful for stratifying patients with respect to current or new lung adenocarcinoma therapies.

In the present study, we explored the relevance of MAP17 expression in lung malignancies and its potential role as a predictive biomarker for currently used systemic therapies in the most prevalent lung cancer histological subtype, adenocarcinoma.

## Methods

### Study approval

Written informed consent was provided by all patients. This project was approved by the Research Ethics Committee of the Hospital Universitario 12 Octubre (Madrid, Spain) (CEI 16/297).

All procedures involving animals were approved by the Consejería de Agricultura y Pesca of the Junta de Andalucía (Approval ref.: SSA/SI/MD/pdm) and by Animal Protection of the Comunidad Autónoma de Madrid (Approval ID: PROEX134/16).

### Clinical samples

The present study utilized different NSCLC patient cohorts. The first cohort comprised 248 subjects diagnosed with NSCLC (Additional file 1: Table S1) from the Virgen del Rocio University Hospital (Seville, Spain) who had undergone surgical resection. Tumour samples were sent to the pathology laboratory for diagnosis and were prepared for storage by formalin fixation and paraffin embedding. Samples were haematoxylin/eosin-stained, and tumoural tissue was selected and included in TMAs by a pathologist to perform immunohistochemistry (IHC). The inclusion criteria were as follows: (1) confirmed NSCLC diagnosis; (2) access to patient clinical information, including age, sex, smoking status, TNM stage, diagnosis date, histologic subtype, date of relapse, date of the last revision and status at that time; and (3) availability of tumour tissue obtained by surgical resection for immunohistochemistry.

A second independent cohort containing 40 DNA samples from patients diagnosed with NSCLC from the Virgen del Rocio University Hospital (Seville, Spain) who had undergone surgical resection was also utilized. These samples were composed of 20 matched tumour and non-tumour samples, each from the same patient. Clinical features of patients with NSCLC for this cohort are summarized in Additional file 1: Table S2 [17]. The majority of samples were obtained from patients following surgical resection for clinical early-stage NSCLC, but ten samples included stages III and IV disease. A description of this cohort can also be found in the literature [18]. During the surgical procedure, the tumour and matched non-tumour tissue samples were collected from patients and immediately snap-frozen at − 80 °C for future use.

A third independent cohort involved 42 subjects diagnosed with advanced (stage III-IV) adenocarcinoma from the University Hospital 12 de Octubre (Madrid, Spain) given erlotinib or gefitinib as first- or subsequent-line treatment. Tumour samples were sent to a pathology laboratory for diagnosis and prepared for storage by formalin fixation/paraffin embedding. The inclusion criteria were as follows: (1) confirmed NSCLC diagnosis; (2) access to patient clinical information; and (3) availability of tumour tissue obtained by surgical resection. Baseline characteristics of patient cohorts are summarized in Additional file 1: Table S3.

For the tumour marker prognostic study, the REMARK [18] reporting guidelines were followed.

### Public databases of clinical samples

To validate our results, we obtained data from publicly available clinical and genomic information from Oncomine (https://powertools.oncomine.com) and TCGA Research Network (http://cancergenome.nih.gov/).

### Cell lines

Twelve lung adenocarcinoma cell lines (A549, H460, H2009, H358, H1650, H1975, HCC827, H2228, H3122, H1437, H1781 and Calu-3), four lung squamous cell carcinoma cell lines (Calu-1, HTB59, H520 and H226) and two immortalized lung epithelial cell lines (NuLi-1 and NL-20) were used (Additional file 1: Table S4) for in vitro studies. All cell lines were purchased from ATCC immediately before beginning this work, with the exception of H3122, which was kindly provided by Dr. Maina. All cell lines were cultured according to ATCC or donor guidelines. All cell lines were regularly tested for mycoplasma.

### Immunohistochemistry

Tumoural area from the samples was identified by pathologists following haematoxylin-eosin staining [19, 32]. Tissue microarrays (TMAs) were constructed from the preselected tumoural area from every biopsy. Tissue processing was performed while protecting samples from oxidation and maintaining the integrity of each sample during the process. De-paraffinization and antigenic epitope recovery was performed using the PTLinK kit (Dako). Immune detection was performed with MAP17 as described [11, 20].

### Illumina 450 K methylation and data processing

The Illumina Infinium Human Methylation 450 BeadChip (Illumina Inc., San Diego, CA, USA) was used to interrogate 485,000 methylation sites per sample across the genome at single-nucleotide resolution. We treated 500 ng of DNA with sodium bisulphate using the EZ DNA Methylation™ Kit and cleaned the DNA with the ZR-96 DNA Cleanup Kit™ (EZ DNA, Zymo Research, Irvine, CA, USA) before standard Illumina amplification, hybridization, and imaging. The resulting intensity files were analysed with Illumina’s GenomeStudio, which generated β-scores. Methylome data were processed as in [17, 20].

### Transfections

Using TransIT-X2 (Mirus), cell lines were transfected with MAP17 overexpression or downregulation plasmids reported in [15, 20, 21].

### Patient-derived xenografts (PDXs) and in vivo treatments

We used a collection of NSCLC PDX models established at the Institute of Biomedicine in Seville (IBIS). Resected lung tumours from HUVR (Hospital Universitario Virgen del Rocío, Sevilla, Spain) patients were obtained from the Biobank of this hospital and subcutaneously inoculated and expanded in successive groups of nude mice to generate a bank for each tumour. For this study, PDXs were selected based on histology, genetic background and MAP17 expression. Tumours were cut into 50–100 mm3 pieces and inserted subcutaneously into one flank of the mouse. Four to eight mice were included in each group. Tumours were measured twice a week, and when tumour volume reached 150 mm3, mice were randomized into different control and treatment groups. Mice were sacrificed at the end of treatment (after 5 weeks), and tumours were harvested. Carboplatin was intraperitoneally administered twice a week at 25 mg/kg/day. Erlotinib was administered five times a week, at 50 mg/kg/day by oral gavage. Bortezomib was administered 5 times a week, at 1 mg/kg/day as in [16]. Mice were monitored daily for signs of distress and were weighed twice a week. Tumour size was determined using callipers according to the following equation: tumour volume = [length Å~ width2]/2. The duration of treatment was 5 weeks, unless rapid tumour growth necessitated an earlier endpoint. In these experiments, blinding was achieved by having one person treat the animals and a different person measure the tumours and process data. All the procedures involving animals were approved by the Consejería de Agricultura y Pesca of the Junta de Andalucía (Approval ref.: SSA/SI/MD/pdm) and by Animal Protection of the Comunidad Autónoma de Madrid (Approval ID: PROEX134/16).

### Immunoblotting

Protein was extracted using RIPA buffer (Sigma) with a protease inhibitor cocktail (cOmplete Mini EDTA-free, Roche) and a phosphatase inhibitor cocktail (PhosSTOP EASYpack, Roche) [21]. We used the following primary antibodies: anti–NFKB-p65 (1:2000; Abcam #ab16502), anti–NFKB-p65 (phospho-Ser536) [93H1] (1:1000; Cell Signaling Technology #3033), anti-IKKa [Y463] (1:5000; Abcam #ab32041), anti-IKKa (phospho-Ser32/36) [5A5] (1:1000; Cell Signaling Technology #9246), anti-p44/4S2 MAPK [137F5] (1:2000; Cell Signaling Technology #4695), anti-p44/42 MAPK (phospho-Thr202/Tyr204) [E10] (1:2000; Cell Signaling Technology #9106), anti-LC3B (1 mg/mL; Abcam #ab48394), EGFR (#4267, CST), pEGFR (#2234, CST) and β-actin (#A5316, Sigma). β-actin protein expression was used as a loading control. Horseradish peroxidase-conjugated secondary antibodies were used for chemiluminescence-based detection of protein expression in the ChemiDoc detection system (BioRad).

### Cell line treatments

The inhibitory concentration 50 (IC50) was calculated as in [22]. For cell line treatments, cells were treated with their IC50 and 2X IC50 concentration for 24 h, and protein was subsequently extracted.

### RNA extraction and analysis

RNA was extracted from cell lines using Trizol reagent (Life Technologies) [9]. RNA extraction of paraffin-embedded patient and PDX tumour tissue was performed with the RecoverAll Extraction Kit (Life Technologies, #AM1975). RNA samples were reverse transcribed using the TaqMan Reverse Transcription Kit (Life Technologies). Gene expression analysis was performed using TaqMan probes from Life Technologies: Hs00906696_m1 FAM (PDZK1IP) and Hs99999905_m1 FAM (GAPDH). GAPDH was used as a reference gene to normalize expression data.

### Public database of cancer cell line sensitivity to drugs

Information on sensitivity to different chemotherapeutic agents, targeted therapies, and MAP17 mRNA expression was obtained from the Genomics of Drug Sensitivity in Cancer (GDSC) [23].

### Statistical methods

In vitro data are represented as the mean ± standard deviation. Statistical analysis was performed with the SPSS statistical package (v19, IBM). In vitro and in vivo experiments were analysed using an unpaired non-parametric Mann-Whitney’s U or Student’s t tests. *p*-values less than 0.05 were considered significant. The Kaplan-Meier method was used for survival analyses of clinical data and cell line xenograft experiments, with a Cox proportional hazards model to adjust for explanatory variables. Overall survival (OS) was defined as the length of time from the date of diagnosis to the date of the last medical record. Progression-free survival (PFS) was defined as the length of time from the date of diagnosis to the date of relapse. A type II ANOVA was used to analyse differences in survival among groups. To obtain hazard ratio values, the Cox proportional hazards model was used.

## Results

### MAP17 Upregulation is a common feature of lung tumours and is preferentially detected in lung adenocarcinoma

**Fig. 1:**
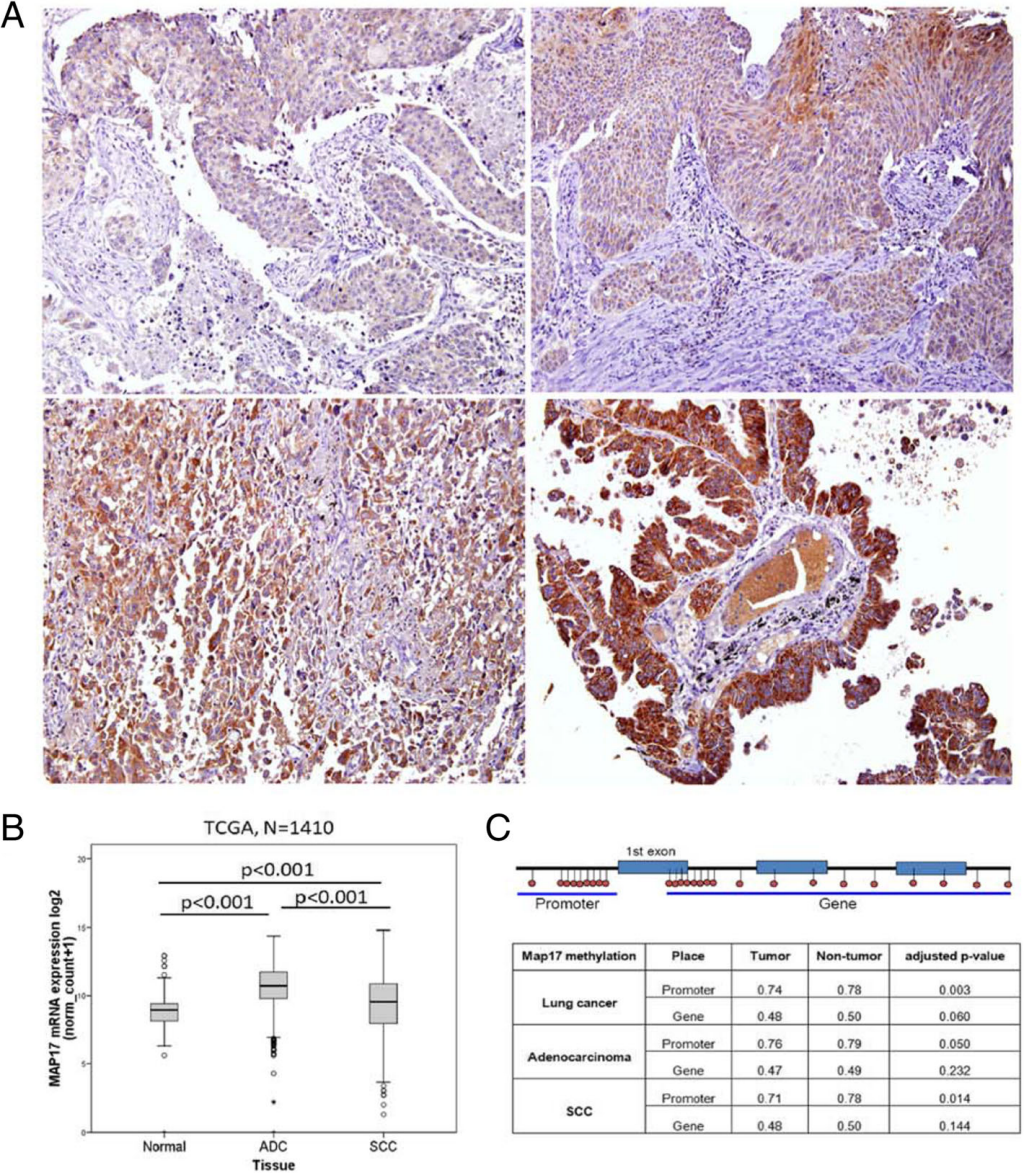
MAP17 upregulation occurs during lung tumourigenesis and is preferentially detected in lung adenocarcinomas. **a** Representative images of MAP17-stained lung tumour samples. **b** MAP17 mRNA expression in non-tumour (Normal), adenocarcinoma (ADC) and squamous cell carcinoma (SCC) samples from the TCGA Lung Cancer cohort. **c** MAP17 promoter and gene methylation in non-tumour and NSCLC samples (See Additional file 1: Table S2). Observed methylation changes (log2 ratio). Statistically significant differences (adjusted *p*-value < 0.05) of methylation levels with respect to those of the control (non-tumour) group were considered. The upper schematic represents the main location of probes for promoter or gene analysis. Below are comparative values of expression

To assess MAP17 expression in the context of lung cancer, we determined MAP17 protein levels in both non-tumour lung tissue and NSCLC samples by immunohistochemistry (Additional file 1: Table S1). We detected increased MAP17 protein expression in tumour samples compared to that in normal lung samples (Fig. 1a). To confirm this result, we examined MAP17 mRNA expression in a TCGA lung cancer patient cohort and observed that this gene is overexpressed in adenocarcinoma and squamous cell carcinoma samples compared to that in normal lung tissue (*p* < 0.001 and p < 0.001, respectively), with adenocarcinomas exhibiting the highest expression levels, superior to even those found in squamous cell carcinoma tumours (p < 0.001, Fig. 1a). These results were further validated in independent cohorts from the publicly available data repository Oncomine (Additional file 1: Figure S1B-D). In addition, MAP17 mRNA levels were determined in a set of non-tumour lung epithelial, adenocarcinoma and squamous cell lung carcinoma cell lines (Additional file 1: Table S4), and similar results were obtained, with adenocarcinoma cell lines exhibiting the highest MAP17 expression levels, followed by squamous cell carcinoma cell lines and non-tumoural cell lines, which showed minimal MAP17 expression (Additional file 1: Figure S1E). Next, to further characterize these results, the methylation profile of MAP17 was evaluated in human lung tumours and compared to that in matched non-tumoural tissues from a cohort of NSCLC patients (Additional file 1: Table S2) using the Illumina Infinium Human Methylation 450 BeadChip. Lung cancer tissue exhibited deregulated MAP17, with lower methylation that was statistically significant in the promoter region (*p* = 0.003). In addition, we identified more notable differences in hypomethylation status at promoter regions in squamous cell carcinoma compared to that in adenocarcinoma (*p* = 0.014 and *p* = 0.050, respectively). Therefore, we detected decreased methylation levels in tumour samples in both adenocarcinoma and squamous cell carcinoma (Fig. 1c), suggesting that MAP17 overexpression in lung cancer may be a consequence of gene demethylation events.

### MAP17 Expression as a prognostic determinant in NSCLC

**Fig. 2:**
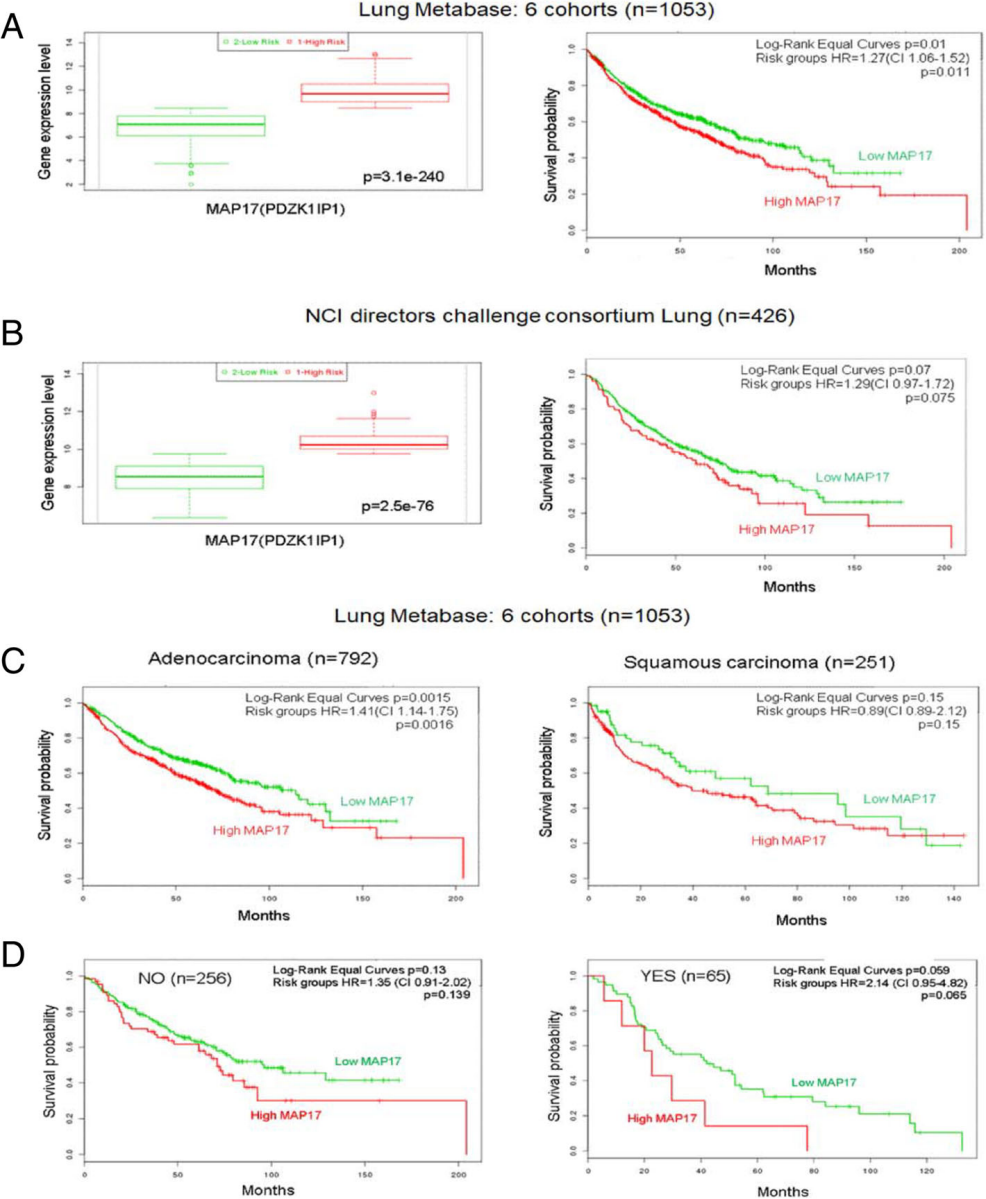
Prognostic analysis of MAP17 expression in NSCLC. **a** Analysis of the risk (left) and survival probability (right) based on MAP17 expression in lung cancer tumours from the Lung Metabase [38]. Red: high risk; Green: low risk. **b** Analysis of the risk (left) and survival probability (right) based on MAP17 expression in lung cancer tumours from the NCI directors challenge consortium database [39]. Red: high risk; Green: low risk. **c** Analysis of survival probability based on MAP17 expression in adenocarcinoma or squamous carcinoma lung cancer tumours from the Lung Metabase database. D) Analysis of survival probability based on adjuvant radiotherapy treatment based on MAP17 expression in lung cancer tumours from the Lung Metabase database (*n* = 1053)

As MAP17 overexpression was observed in NSCLC samples, we decided to assess whether its expression correlates with clinical characteristics in different public transcriptomic datasets (Lung Metabase (six validated cohorts), *n* = 1053, (SurvExpress compilation) and Directors Challenge Consortium NCI lung, *n* = 453, PMID: 18641660). In the Lung Metabase formed by 6 cohorts (n = 1053), we observed that high levels of MAP17 mRNA correlate to high risk for tumours and trend to poor prognosis in total lung tumours (Fig. 2a). Similar data were observed in the lung tumour cohort from Directors Challenge Consortium (*n* = 462) (Fig. 2b). When the former cohort of 1056 tumours was subdivided according to histology, we still observed a clear trend for worse prognosis in both common types of tumours, ADC and SCC (Fig. 2c**).** Furthermore, this trend for worse prognosis was independent of stage (Additional file 1: Figure S2).

Interestingly, when the individuals were separated by radiation treatment, the individuals with increased levels of MAP17 still showed worse prognosis (Fig. 2d), indicating the relevance of the determination of this marker in the response.

Overall, taken together, the data suggest that high MAP17 levels showed worse prognosis than did patients with tumours with low MAP17 levels, and this is independent of histology and stage and radiation treatment. Therefore, we explored whether MAP17 can be a marker for specific chemotherapy responses and whether we can improve them.

### MAP17 Expression is predictive for platinum-based therapy sensitivity in lung adenocarcinoma

**Fig. 3:**
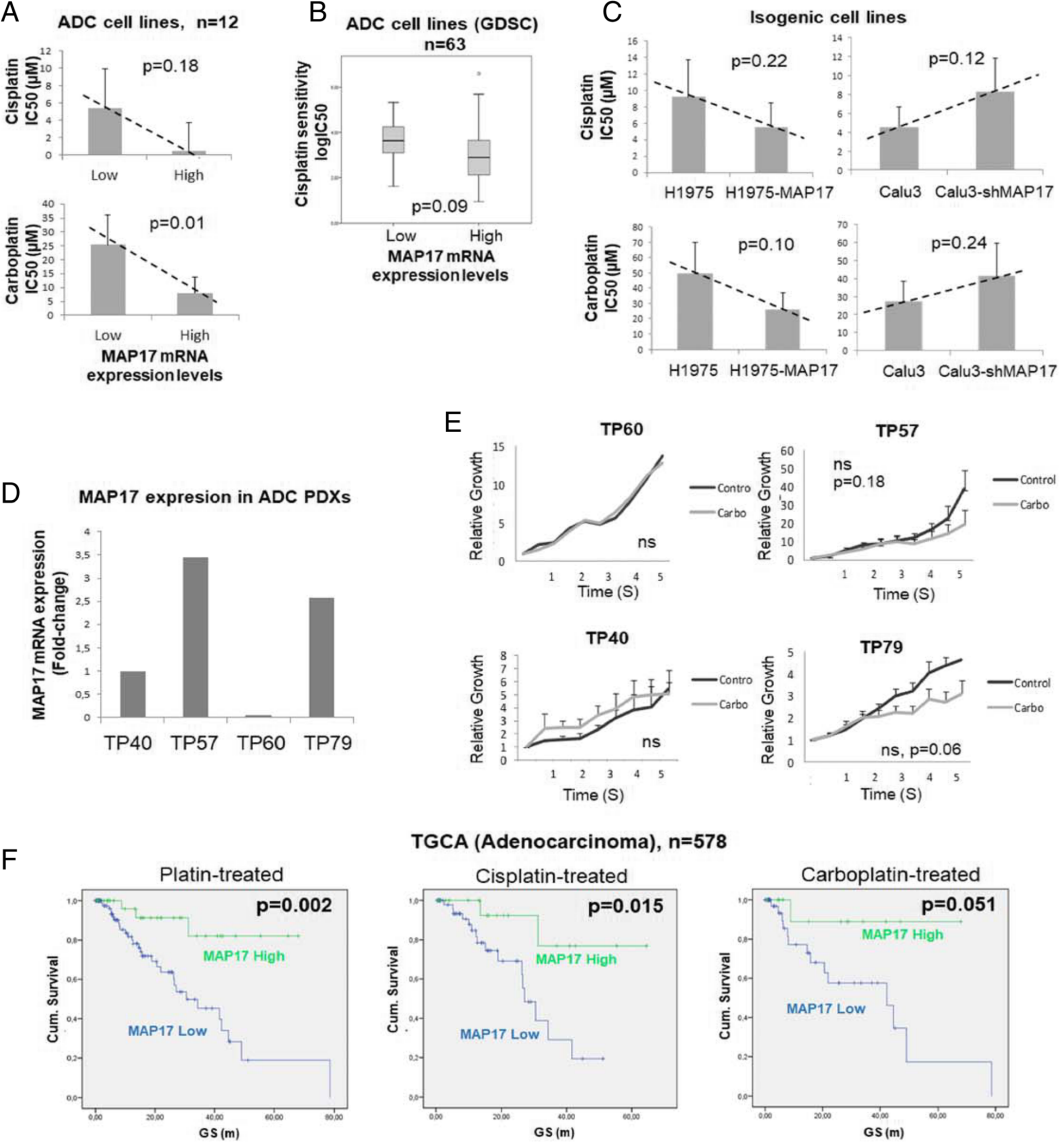
MAP17 expression predicts platin sensitivity in lung adenocarcinoma. **a** Correlation of cisplatin (up) and carboplatin (down) sensitivity in lung adenocarcinoma cell lines based on MAP17 expression. **b** Correlation of cisplatin sensitivity in adenocarcinoma cell lines based on MAP17 expression, with data obtained from the GDSC database. **c** Cisplatin (up) and carboplatin (down) sensitivity in MAP17-overexpressing and MAP17-silenced lung adenocarcinoma cell lines. **d** Determination of MAP17 mRNA expression in lung adenocarcinoma PDXs. **e** Tumour growth assessment of carboplatin-treated adenocarcinoma PDXs with differential MAP17 expression. **f** Kaplan-Meier overall survival curves for lung adenocarcinoma patients from the TCGA database (Additional file 1: Table S6) treated with cisplatin, carboplatin, or both. Patients were divided based on MAP17 expression levels into two groups, below or above the 75th percentile of expression

As MAP17 expression was highest in adenocarcinoma (Additional file 1: Figure S1), which is the lung tumour subtype with the highest prevalence, we decided to focus our work on this histological subtype. As MAP17 has been correlated with platinum-based compounds through ROS induction in other tumour types [10, 11], we evaluated sensitivity to cisplatin and carboplatin, the gold-standard therapy in most of the advanced lung cancer cases, in a panel of 13 adenocarcinoma cell lines (Additional file 1: Table S5). To this aim, we determined the drug concentration inhibiting 50% of cell growth (IC50) and correlated these data with MAP17 mRNA levels. We observed a clear trend showing that cell lines with high MAP17 expression were more sensitive to cisplatin (*p* = 0.18) and a statistically significant association of higher MAP17 levels and carboplatin sensitivity (*p* = 0.01, Fig. 3a). Furthermore, we confirmed these results for cisplatin by analysing publicly available data from the Genomics of Drug Sensitivity in Cancer (GDSC) database. We found the same trend in the database, suggesting that adenocarcinoma cell lines with high MAP17 expression are more sensitive to this compound (*p* = 0.09, Fig. 3b). In addition, we overexpressed MAP17 in the adenocarcinoma cell line H1975, which normally expresses low levels of MAP17, and silenced expression of this gene in the Calu3 line, which normally expresses high levels of MAP17. We then assessed the sensitivity of these cell lines to the platinum compounds. We observed higher sensitivity to both drugs in cell lines with high levels of MAP17 and low sensitivity to the drugs in cells with low levels of MAP17 (Fig. 3c), further supporting our previous results.

To assess the in vivo relevance of these findings, we assessed MAP17 mRNA expression in four lung adenocarcinoma PDXs (Fig. 3d) and treated them with carboplatin. We observed that the two models exhibiting low MAP17expression, TP40 and TP60, were insensitive to carboplatin treatment, while those models with the highest MAP17 expression, TP57 and TP79, were sensitive to the drug, with tumour growth inhibition (TGI) percentages of 49.5% and 66.4%, respectively (Fig. 3e).

Finally, to assess the predictive potential of MAP17 expression for platinum-based therapy response, we analysed survival of a subset of patients from the TCGA adenocarcinoma cohort treated with cisplatin and/or carboplatin. We found that patients with high MAP17expression exhibited higher overall survival when analysing cisplatin/carboplatin-, cisplatin- and carboplatin-treated subsets (*p* = 0.002, *p* = 0.015 and *p* = 0.051, respectively, Fig. 3f).

### MAP17 Expression predicts response to EGFR inhibitors in lung adenocarcinoma

**Fig. 4:**
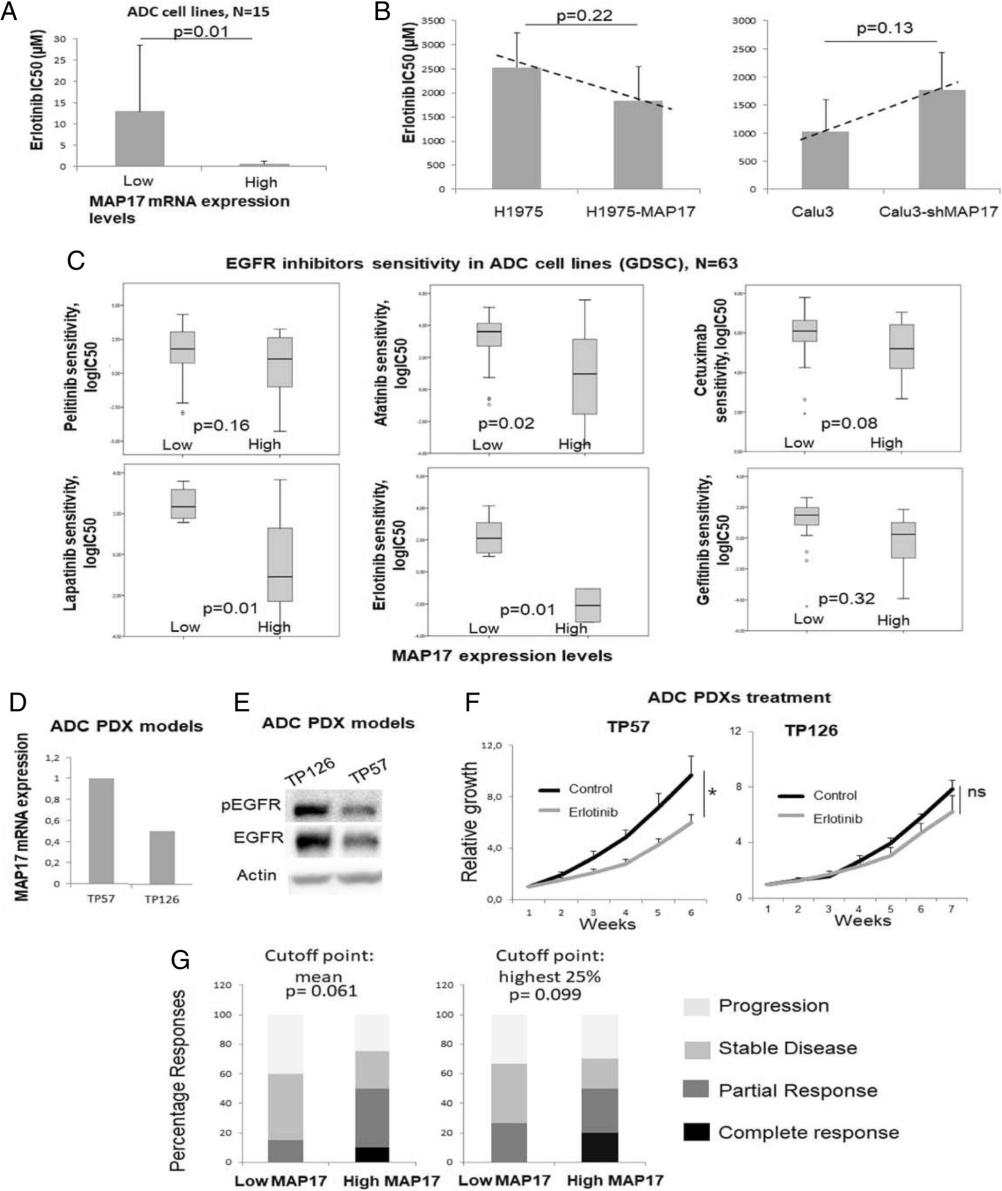
MAP17 expression predicts sensitivity to erlotinib in lung adenocarcinoma. **a** Erlotinib sensitivity in lung adenocarcinoma cell lines based on MAP17 mRNA expression. **b** Erlotinib sensitivity in one MAP17-overexpressing and one MAP17-silenced lung adenocarcinoma cell lines. **c** Analysis of sensitivity to different EGFR inhibitors in lung adenocarcinoma cell lines based on MAP17 expression using data from the GDSC database. Determination of MAP17 expression (**d**) and EGFR activation (**e**) in lung adenocarcinoma PDXs. (F) Tumour growth assessment in erlotinib-treated adenocarcinoma PDXs with differential MAP17 expression. **g** Response to erlotinib or gefitinib treatment in adenocarcinoma patients (Additional file 1: **Table S3**) with tumours harbouring low or high MAP17 mRNA expression. Expression was considered as high with values above the median value (left) or the 75th percentile (right)

As EGFR inhibitors have been associated with ROS induction, similar to platinum-based compounds [24], we assessed the predictive potential of MAP17expression for erlotinib sensitivity in vitro. To this aim, we determined erlotinib IC50 in our adenocarcinoma cell line panel and compared those results to the MAP17 levels (Additional file 1: Tables S4 and S5). We found that cell lines with the highest MAP17 expression showed increased erlotinib sensitivity compared to that of the other the cell lines (p = 0.01, Fig. 4a). In addition, we determined erlotinib sensitivity in MAP17-overexpressing H1975 and MAP17-silenced Calu-3 cell lines and found a trend suggesting that MAP17 overexpression correlated with increased erlotinib sensitivity (*p* = 0.22), while MAP17 downregulation was associated with higher resistance to this tyrosine kinase inhibitor (TKI) (*p* = 0.13, Fig. 4b). To confirm these results, we analysed data concerning adenocarcinoma cell line sensitivity to different EGFR inhibitors from the GDSC database. We found that high MAP17 expression correlated with increased sensitivity to six different EGFR inhibitors: pelitinib (*p* = 0.16), afatinib (*p* = 0.02), cetuximab (*p* = 0.08), lapatinib (*p* = 0.32), erlotinib (*p* < 0.01) and gefitinib (p < 0.01) (Fig. 4c).

To further support our findings, we assessed the mRNA expression of MAP17 and EGFR in different adenocarcinoma PDXs (Fig. 4d, e) and treated them with erlotinib. Two PDX models with expression of high levels of wild-type EGFR, TP57 and TP126 were selected for these experiments. We observed that the model with low MAP17 expression (TP126) showed no sensitivity to erlotinib treatment, while the model with high MAP17 levels exhibited sensitivity to this EGFR TKI, with a TGI rate of 67% (*p* = 0.048, Fig. 4f).

Finally, we examined our cohort of patients treated with erlotinib (Additional file 1: Table S3) and analysed the response according to MAP17 levels. When we used the mean as the cutoff point (Fig. 4g), we found a clear increase in partial responses, and a small percentage of complete responses appeared. In total, we observed 50% responders vs 15% in the lowest MAP17 expressors. Furthermore, when we analysed the 25% patients with tumours expressing the highest levels of MAP17, we observed 50% responders vs 25% in low-MAP17 expressors (Fig. 4g); however, the complete responses rate rose to 20%.

Taken together, these data clearly indicate that the MAP17 level is an independent marker that predicts the response to erlotinib treatment in lung adenocarcinoma patients.

### MAP17 Expression levels predict efficacy of the proteasome inhibitor bortezomib in lung adenocarcinoma

**Fig. 5:**
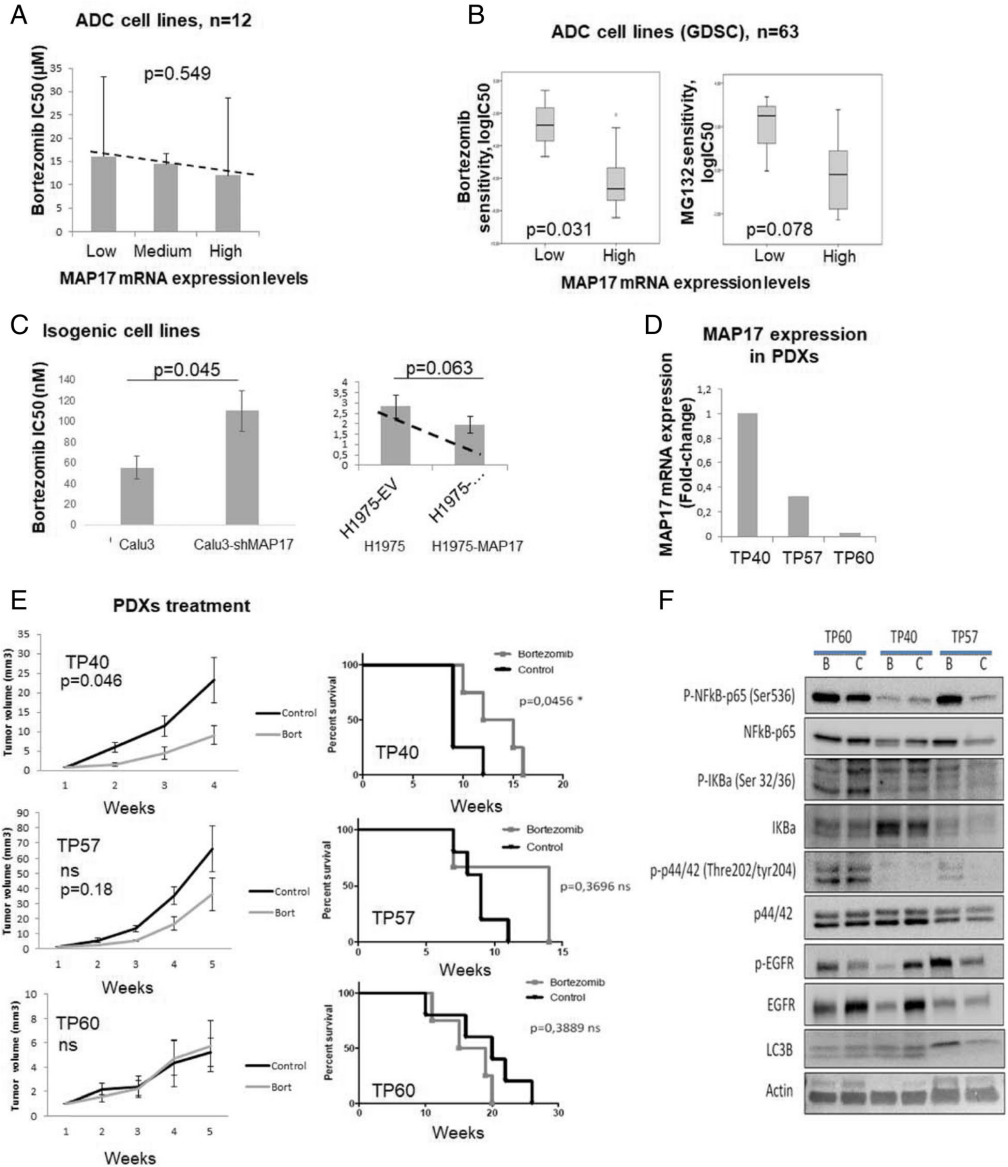
MAP17 expression predicts sensitivity to bortezomib in lung adenocarcinoma. **a** Bortezomib sensitivity in low-, medium- and high-MAP17-expressing lung adenocarcinoma cell lines. **b** Sensitivity of low- and high-MAP17-expressing adenocarcinoma cell lines to bortezomib and to another proteasome inhibitor, MG132, obtained from the GDSC database. **c** Bortezomib sensitivity in the Calu-3 lung adenocarcinoma cell line after MAP17silencing and in the H1975 lung adenocarcinoma cell line after MAP17 overexpression. **d** Assessment of MAP17 mRNA expression in adenocarcinoma PDX models. **e** Tumour growth assessment (left) and survival analysis (right) in three bortezomib-treated adenocarcinoma PDXs with differential MAP17 expression. **f** Western blot showing activation of oncogenic signalling- and autophagy-related pathways in bortezomib-treated and control adenocarcinoma PDX models

Despite our promising findings with platinum-based therapies and EGFR inhibitors, there is still a high percentage of patients who may not respond to these therapies [4, 22], highlighting the necessity for novel therapies in lung cancer. We previously demonstrated that the proteasome inhibitor bortezomib shows efficacy in high-MAP17-expressing malignancies from different origins, including sarcomas and breast tumours [14, 15]. To study the efficacy of this drug in the context of lung adenocarcinoma, we determined sensitivity to bortezomib in our lung adenocarcinoma cell line panel (Additional file 1: Tables S4 and S5). We identified a trend showing that increased MAP17 expression correlated with higher bortezomib sensitivity (Fig. 5a). To confirm these results, we studied the sensitivity to bortezomib and to another proteasome inhibitor, MG132, in adenocarcinoma cell lines from the GDSC database, and we found that high MAP17 expression correlated with higher sensitivity to both proteasome inhibitors (*p* = 0.031 and *p* = 0.078, respectively, Fig. 5b). In addition, we found that either increased MAP17 mRNA levels in the H1975 cell line or MAP17 silencing in the high-MAP17-expressing lung adenocarcinoma Calu-3 cell line both resulted in reduced sensitivity to bortezomib (*p* = 0.063, *p* = 0.045, respectively, Fig. 5c).

To assess the efficacy of bortezomib in vivo, we selected three lung adenocarcinoma PDXs with differential MAP17 expression levels (TP40, TP57 and TP60, Fig. 5d). Upon bortezomib treatment, the high-MAP17-expressing PDX model TP40 showed a high response to the proteasome inhibitor, while the medium-MAP17-expressing TP57 model showed a more modest response, and the low-MAP17-expressing model TP60 showed no sensitivity to bortezomib (Fig. 5e), consistent with our in vitro results.

We previously demonstrated that MAP17 prevents cytoprotective NFκB activation and autophagy induced by bortezomib in both breast and sarcoma tumour cells [14, 15]. Therefore, we next tested whether these molecular markers might explain the response to bortezomib in our PDX models (Fig. 5f). The high-MAP17-expressing PDX model TP40 that responds to bortezomib showed lower levels of endogenous active NFκB (measured as p65 phosphorylated at Ser536 and high IKBa), confirming the mechanistic role of this factor in the bortezomib response. Interestingly, lower levels of both EGFR activation and its downstream effector pERK were also observed. This inhibition of the ERK pathway might contribute to decreased anti-apoptotic signals, thereby increasing the effect of bortezomib. The effect on the low-MAP17-expressing model TP60 was the opposite (Fig. 5f), with high NFKB activation as well as high EGFR and low autophagy, all signals contributing to block apoptosis in response to bortezomib treatment. Interestingly, the MAP17-expressing TP57 model with medium expression of MAP17 showed a partial response to bortezomib. The TP57 model also exhibited partial activation of the abovementioned pathways (Fig. 5f**)**. For instance, under bortezomib treatment, the NFKB levels are decreased in the controls, but with the high-expressing MAP17 TP40 model, these levels increase. Autophagy is not altered, but MAPK activation is clearly diminished.

Therefore, combined inhibition of NFKb, autophagy and EGFR protective pathways induced by MAP17 may explain the increase in sensitivity to bortezomib observed in vivo. The differing activity of each pathway is very likely molecule- and context-dependent for each tumour.

## Discussion

We have shown that MAP17 expression is induced in lung tumourigenesis, particularly in adenocarcinomas, mainly by demethylation, and that it correlates with higher tumour stage. In addition, we provide evidence that MAP17 levels predict sensitivity to therapies currently under clinical use, including platinum-based compounds and EGFR inhibitors, and that treatment with proteasome inhibitors, especially bortezomib, may be a novel therapeutic approach for MAP17-overexpressing adenocarcinomas.

MAP17 upregulation is a common feature in tumours from diverse histological origin, including cervical, breast, prostate, and ovarian, and is correlated with tumour progression [7–11, 20, 25]. We show that these observations may extend to lung carcinomas, which show increased MAP17 expression compared to that of normal lung tissue, especially in lung adenocarcinomas, the histological subtype with the highest MAP17 levels. Furthermore, we provide evidence that this upregulation may be a consequence of MAP17 promoter and gene demethylation. We also observed that MAP17 expression is higher in tumours with higher stage in two independent cohorts. Furthermore, we found a clear association between MAP17 mRNA levels and worse prognosis in two independent cohorts, the Lung Metabase dataset comprising six different lung cancer cohorts (1056 samples) and the NCI Directors Challenge Consortium (462 samples). This prognosis extends to tissue specificity. Therefore, we suggest a prognostic role for MAP17 expression in NSCLC wherein high levels of MAP17 indicate poor prognosis in NSCLC.

MAP17 exerts pro-tumourigenic effects in tumour cells by increasing ROS levels [7]. However, increased ROS may represent an Achilles’ heel for tumour cells in contexts where ROS production and apoptosis are over-promoted, as exposure to platinum-based compounds may be more effective [26, 27]. Correlating with this hypothesis, high MAP17 expression is associated with increased survival in patients treated with cisplatin in several tumour settings [10, 11]. In line with these results, we provide in vitro*,* in vivo and clinical evidence that, in the context of lung adenocarcinoma, MAP17 levels may be a potential predictive biomarker for platinum-based chemotherapy. Therefore, determination of expression levels of this gene may help select patients who will benefit from this type of therapy.

ROS induction has been related to other treatments, including EGFR inhibitors [24, 28], so we examined whether MAP17 expression can predict the response to EGFR-targeted therapy. We found that high MAP17 expression correlates with increased sensitivity to a variety of EGFR inhibitors in vitro and with increased sensitivity to erlotinib in lung adenocarcinoma PDX models with high EGFR activation. EGFR inhibitors are the current standard of care for adenocarcinoma patients with EGFR activating mutations. However, 10–15% of these patients do not respond to this therapy, highlighting the necessity for predictive biomarkers to identify resistant tumours [29]. Additionally, the EGFR inhibitor erlotinib was shown to prolong survival in unselected NSCLC patients after first- or second-line chemotherapy, suggesting that some wild-type EGFR tumours may be sensitive to EGFR inhibition [30]. In fact, our assessment of MAP17 levels in erlotinib-treated patients indicates that high levels of MAP17 are indicative of better response rates and even complete responses. Therefore, MAP17 assessment could help select patients who may benefit more so from EGFR inhibition therapy.

Unfortunately, despite demonstration of efficacy and approval for clinical use of both targeted treatments and immunotherapies in the lung adenocarcinoma setting, a significant number of patients harbour tumours unresponsive to these treatments [4, 5, 29], leaving them with very limited therapeutic options. The proteasome inhibitor bortezomib, which has been approved by the FDA for the treatment of multiple myeloma and mantle cell lymphoma [12, 13], has been shown as a promising treatment for high-MAP17-expressing tumours from different origins in preclinical studies [14, 15]. In light of these results, we examined whether these findings may be extended to lung adenocarcinomas. We found that high MAP17 levels are linked to bortezomib sensitivity in our adenocarcinoma cell lines, confirming these results for bortezomib and an alternative proteasome inhibitor in a publicly available database. Furthermore, we found that MAP17 expression predicts bortezomib response in lung adenocarcinoma PDXs. These results supported the potential efficacy of bortezomib in high-MAP17 expressing lung adenocarcinomas. These data are similar to those obtained for breast carcinoma and sarcoma [14, 15]. In fact, analysis of multiple myeloma patients also suggests that high levels of MAP17 are indicative of a better response [14, 15]. We previously found that MAP17 overexpression induces a clear increase in sensitivity to bortezomib in mammary carcinoma and sarcoma cells, which was reproduced both in vitro and in vivo [14, 15, 31, 32]. The MAP17-dependent increase in sensitivity correlated with inhibition of the cytoprotective effect induced by nuclear translocation of phosphorylated NFκB and autophagy induced by bortezomib [14, 15, 31, 32].

Bortezomib-induced NFκB phosphorylation promotes its translocation to the nucleus and its activity as a transcription factor. NFκB nuclear translocation activates anti-apoptotic genes and promotes survival [33–35]. High levels of MAP17 reduce NFκB nuclear translocation in response to bortezomib treatment, reducing its anti-apoptotic role in tumours. Therefore, the presence of MAP17 renders tumour cells unable to exploit these cytoprotective effects [31, 32]. In contrast, it is generally thought that autophagy has two opposing functions in tumour cells in response to chemotherapy-induced stress: cytoprotection and cytotoxicity [36, 37]. Bortezomib also induced an initial cytoprotective role by inducing autophagy that was reduced by the presence of MAP17, increasing the cytotoxic response to the drug [31, 32].

Ultimately, high levels of MAP17 could be of prognostic value for predicting treatment response in patients with diseases clinically treated with bortezomib. Additionally, high MAP17 levels could be used to select patients with other tumours for which bortezomib is not currently an indication.

## Conclusions

Our results suggest a prognostic role for MAP17 in lung tumours, with particular relevance in adenocarcinomas. MAP17 levels may convey predictive potential for platinum-based therapy and EGFR inhibitor efficacy. Furthermore, we propose bortezomib treatment as a novel efficacious therapy for adenocarcinomas exhibiting high MAP17 expression.

## Additional file

Additional file 1: **Table S1.** Clinicopathological characteristics of the NSCLC cohort 1 from which tumor samples were analyzed by Immunohistochemistry. **Table S2**. Clinicopathological characteristics of the NSCLC cohort from which frozen tumor tissue was analyzed. Used for the study of methylation of MAP17 promoter and gene. Cohort number 2. **Table S3.** Clinicopathological characteristics of the erlotinib/gefitinib-treated NSCLC patient cohort. Cohort number 3. **Table S4.** Description of the driver molecular alterations and MAP17 mRNA expression of our lung cell line panel **Figure S1**. Related to Fig. 1. ADC=Adenocarcinoma, SCC=Squamous Cell Carcinoma, TN=“Triple Negative” (referring to the absence of alterations in KRAS, EGFR or ALK), I=Immortalized. **Table S5.** IC50 sensitivity values of adenocarcinoma cell lines to cisplatin, carboplatin, erlotinib and bortezomib. **Table S6.** clinicopathological characteristics of the platinum-treated lung adenocarcinoma TCGA cohort**. Figure S1.** MAP17 upregulation occurs during lung tumorogenesis and is preferentially detected in lung adenocarcinomas. (A-D) MAP17 mRNA expression in non-tumor and NSCLC samples of different histologic subtypes from different publicly available databases accessible at Oncomine (https://powertools.oncomine.com). NT lung = Lung non-tumoral tissue, LCLC = Large cell carcinoma. (E) MAP17 mRNA expression in lung epithelial immortalized non-tumoral (normal), adenocarcinoma (ADC) and squamous cell carcinoma (SCC) cell lines. **Figure S2.** Analysis of the survival probability according to MAP17 expression in differeng grades or stage of Lung cancer tumors in the Lung Metabase database (n=1053). **Figure S3.** Relationship between MAP17 mRNA levels and EGFR mutations (based on Table S5). (DOCX 411 kb)

## Abbreviations

ADC: adenocarcinoma
ALK: anaplastic lymphoma kinase
DD96: differential display gene 96 (same as Map17)
EGFR: Epidermal growth factor receptor
ERK: extracellular signal-regulated kinase (same as MAPK)
FDA: Food and Drug Administration
GAPDH: glyceraldehyde-3-phosphate dehydrogenase
GDSC: Genomics of Drug Sensitivity in Cancer
IC50: inhibitory concentration 50%
IHC: Immunohistochemistry
IKKa: I-kappa-b kinase a
LCC: large cell carcinoma
MAP17: Membrane-associated protein of 17 kDa
MAPK: mitogen-activated protein kinase
NFKB: nuclear factor kappa-b
NSCLC: non-small cell lung cancer
OS: Overall survival
PDX: Patient direct xenograft
PDZK1IP1: PDZK1-interacting protein 1, (PDZK1, PDZ domain-containing 1)
PFS: Progression-free survival
ROS: reactive oxygen species
SCC: squamous cell carcinoma
SCLC: small cell lung cancer
TCGA: The Cancer Genome Atlas
TKI: tyrosine kinase inhibitor
TMA: Tissue microarray
