# Supplementary material for: MAP17 predicts sensitivity to platinum-based therapy, EGFR inhibitors and the proteasome inhibitor bortezomib in lung adenocarcinoma
Source: J Exp Clin Cancer Res. 2018 Aug 17;37:195. doi: 10.1186/s13046-018-0871-7 (PMC6098621; doi:10.1186/s13046-018-0871-7)
Supplement: Supplementary file 1 — Table S1. Clinicopathological characteristics of the NSCLC cohort 1 from which tumor samples were analyzed by Immunohistochemistry. Table S2. Clinicopathological characteristics of the NSCLC cohort from which frozen tumor tissue was analyzed. Used for the study of methylation of MAP17 promoter and gene. Cohort number 2. Table S3. Clinicopathological characteristics of the erlotinib/gefitinib-treated NSCLC patient cohort. Cohort number 3. Table S4. Description of the driver molecular alterations and MAP17 mRNA expression of our lung cell line panel Figure S1. Related to Fig. 1. ADC=Adenocarcinoma, SCC=Squamous Cell Carcinoma, TN=“Triple Negative” (referring to the absence of alterations in KRAS, EGFR or ALK), I=Immortalized. Table S5. IC50 sensitivity values of adenocarcinoma cell lines to cisplatin, carboplatin, erlotinib and bortezomib. Table S6. clinicopathological characteristics of the platinum-treated lung adenocarcinoma TCGA cohort. Figure S1. MAP17 upregulation occurs during lung tumorogenesis and is preferentially detected in lung adenocarcinomas. (A-D) MAP17 mRNA expression in non-tumor and NSCLC samples of different histologic subtypes from different publicly available databases accessible at Oncomine (https://powertools.oncomine.com). NT lung = Lung non-tumoral tissue, LCLC = Large cell carcinoma. (E) MAP17 mRNA expression in lung epithelial immortalized non-tumoral (normal), adenocarcinoma (ADC) and squamous cell carcinoma (SCC) cell lines. Figure S2. Analysis of the survival probability according to MAP17 expression in differeng grades or stage of Lung cancer tumors in the Lung Metabase database (n=1053). Figure S3. Relationship between MAP17 mRNA levels and EGFR mutations (based on Table S5). (DOCX 411 kb) [file 13046_2018_871_MOESM1_ESM.docx]

**Additional file**

**Table S1**. Clinicopathological characteristics of the NSCLC cohort 1 from which tumor samples were analyzed by Immunohistochemistry.

| **Variable** | **NSCLC (N=248)** |
| --- | --- |
| **Gender**  Male  Female | 233(94.0%)  15(6.0%) |
| **ECOG Performance status**  0  1  2 | 167(67.3%)  72(29.0%)  3(1.2%) |
| **Age** | 66 [39-84] |
| **Smoking habits**  Ex smoker  Current smoker  Never smoker | 113(45.6%)  120(48.4%)  11 (4.4%) |
| **Histology**  Squamous cell carcinoma  Adenocarcinoma  Large cell carcinoma  Other | 123 (49.6%)  77 (31.0%)  22 (8.9%)  26 (10.4%) |
| **Stage**  IA  IB  IIA  IIB  IIIA | 27 (10.9%)  114 (46.0%)  4 (1.6%)  65 (26.2%)  37 (14.9%) |
| **Tumour differentiation**  Well differentiated  Moderately differentiated  Poorly differentiated | 18 (7.3%)  78 (31.5%)  98 (39.4%) |
| **Adjuvant radiotherapy**  Yes  No | 30 (12.1%)  205 (82.7%) |
| **Adjuvant chemotherapy**  Yes  No | 24 (9.7%)  213 (85.9%) |
| **Relapse**  Yes  No | 119 (48.0%)  109 (44.0%) |
| **Exitus**  Yes  No | 153 (61.7%)  76 (30.6%) |
| **Reason for Exitus**  Progression  Not related  Undetermined  Surgical complications  Toxicity  Second tumour | 104 (41.9%)  22 (8.9%)  107 (43.1%)  4 (1.6%)  10 (4.0%)  1 (0.4%) |

Continuous variables are expressed as median [interquartile range] and categorical variables are expressed as the number of cases (percentage).

**Table S2**. Clinicopathological characteristics of the NSCLC cohort from which frozen tumor tissue was analyzed. Used for the study of methylation of MAP17 promoter and gene. Cohort number 2.

| \|  \| **NSCLC (N=40)** \| \| --- \| --- \| \| **Gender** \|  \| \| Male \| 90 (36) \| \| Female \| 10 (4) \| \| **Age (years)** \| 67 [60-73] \| \| **Smoking status** \|  \| \| Smokers \| 40.4 (19) \| \| Ex-smokers \| 44.7 (21) \| \| **Packs-year** \| 41.0 [20.0-65.7] \| \| **Histology** \|  \| \| Adenocarcinoma \| 50 (20) \| \| Squamous cell carcinoma \| 50 (20) \| \| **Staging** \|  \| \| Stage I \| 40 (16) \| \| Stage II \| 37.5 (15) \| \| Stage III-IV \| 22.5 (9) \| |
| --- | --- | --- | --- | --- | --- | --- | --- | --- | --- | --- | --- | --- | --- | --- | --- | --- | --- | --- | --- | --- | --- | --- | --- | --- | --- | --- | --- | --- | --- | --- | --- | --- |

Continuous variables are expressed as the median [interquartile range (IQR)] and categorical variables are expressed as the number of cases (%).

**Table S3.** Clinicopathological characteristics of the erlotinib/gefitinib-treated NSCLC patient cohort. Cohort number 3

| **Variable** | **Adenocarcinoma (N=42)** |
| --- | --- |
| **Gender**  Male  Female | 22 (52.42%)  20 (47.6%) |
| **Age** | 67 [50-84] |
| **Stage**  IIIA  IIIB  IV | 1 (2.4%)  1 (2.4%)  40 (95.2%) |
| **EGFR mutational status**  Wild type  Mutated  Unknown | 17 (40.5%)  22 (52.4)  3 (7.1%) |
| **Drug**  Erlotinib  Gefitinib | 28 (66.7%)  14 (33.3%) |
| **Adjuvant chemotherapy**  Yes  No | 24 (9.7%)  213 (85.9%) |
| **Line of treatment**  1^st^  2^nd^  ≥3^rd^ | 16 (38.1%)  10 (23.8%)  16 (38.1%) |
| **Response to treatment**  Complete  Partial  Stable disease  Progression  Unknown | 2 (4.8%)  11 (26.2%)  14 (33.3%)  13 (31.0%)  2 (4.8%) |

Continuous variables are expressed as median [interquartile range] and categorical variables are expressed as the number of cases (percentage).

**Table S4.** Description of the driver molecular alterations and MAP17 mRNA expression of our lung cell line panel **Figure S1**. Related to **Figure 1**. ADC=Adenocarcinoma, SCC=Squamous Cell Carcinoma, TN=”Triple Negative” (referring to the absence of alterations in KRAS, EGFR or ALK), I=Immortalized

| **Cell line** | **Histology** | **Driver mutation** | **Reference for driver mutation** | **MAP17 mRNA expression (2^-ÄCt^)** | |
| --- | --- | --- | --- | --- | --- |
|  |  |  |  | **Mean** | **Standard deviation** |
| **A549** | ADC | KRAS p.G12S | (Helfrich, Raben et al. 2006) | 0.00044559 | 8.06E-05 |
| **H460** | ADC | KRAS p.Q61H | (Helfrich, Raben et al. 2006) | 8.69E-05 | 2.34E-05 |
| **H2009** | ADC | KRAS p.G12A | COSMIC | 0.00044716 | 3.33E-05 |
| **H358** | ADC | KRAS p.G12C | (Helfrich, Raben et al. 2006) | 0.00026215 | 7.09E-05 |
| **H1650** | ADC | EGFR E746-E750 del | (Blanco, Iwakawa et al. 2009) | 4.66E-05 | 9.35E-06 |
| **H1975** | ADC | EGFR L858R/T790M | COSMIC | 0.00033741 | 5.66E-05 |
| **HCC827** | ADC | EGFR E746-E750 del | (Helfrich, Raben et al. 2006) | 0.01479002 | 0.0007266 |
| **H3122** | ADC | EML4-ALK v1 translocation | COSMIC | 0.00010772 | 2.35E-05 |
| **H2228** | ADC | EML4-ALK v3 translocation | COSMIC | 0.00305051 | 0.00035168 |
| **H1781** | ADC | TN (*L858R mutation detected in our lab) | (Helfrich, Raben et al. 2006) | 0.00400609 | 0.00033551 |
| **H1437** | ADC | TN | (Helfrich, Raben et al. 2006) | 0.00160354 | 0.00026727 |
| **Calu-3** | ADC | TN | (Helfrich, Raben et al. 2006) | 0.01495963 | 0.00167956 |
| **Calu-1** | SSC | KRAS p.G12C | COSMIC | 0.00022121 | 2.13E-05 |
| **HTB59** | SSC | KRAS p-G12V | COSMIC | 0.00707563 | 0.00063242 |
| **H520** | SSC | TN | COSMIC, (Helfrich, Raben et al. 2006) | 6.98E-05 | 9.72E-06 |
| **H226** | SSC | TN | COSMIC, (Helfrich, Raben et al. 2006) | 2.13E-05 | 2.75E-06 |
| **NL20** | I | TN | COSMIC | 2.26E-05 | 1.80E-06 |
| **NuLi-1** | I | TN | COSMIC | 0.00042171 | 0.00024266 |

**Table S5.** IC50 sensitivity values of adenocarcinoma cell lines to cisplatin, carboplatin, erlotinib and bortezomib.

| **Cell line** | **Cisplatin IC50 (µM)** | **Carboplatin IC50 (µM)** | **Erlotinib IC50 (µM)** | **Bortezomib IC50 (nM)** |
| --- | --- | --- | --- | --- |
| **A549** | 10.34 | 42.15 | 4.45 | 13.37 |
| **Calu-3** | 0.40 | 14.90 | 0.47 | 59.96 |
| **H1437** | 4.39 | 37.88 | 48.49 | 15.65 |
| **H1650** | 6.10 | 19.58 | 24.81 | 4.97 |
| **H1781** | 0.06 | 3.52 | 1.21 | 8.90 |
| **H1975** | 5.35 | 27.55 | 5.34 | 4.98 |
| **H2009** | 15.21 | 17.84 | 25.98 | 21.09 |
| **H2228** | 3.60 | 16.90 | 12.93 | 13.38 |
| **H226** | 4.91 | 36.27 | 14.45 | 12.40 |
| **H3122** | 6.31 | 30.13 | 29.88 | 16.09 |
| **H358** | 0.19 | 25.37 | 0.33 | 16.73 |
| **H460** | 1.96 | 8.47 | 9.57 | 61.10 |
| **HCC827** | 6.03 | 8.12 | 0.01 | 12.07 |

| Variable | Platin-treated ADC TCGA (N=56) |
| --- | --- |
| Gender     Male     Female | 28 (50)  28 (50) |
| Age | 58.6 [53.2-68] |
| Number of packs per year | 27.8 [15-40] |
| Stage     I     II     III     IV  Unknown | 9 (16.1)  24 (42.8)  16 (28.6)  6 (10.7)  1 (1.8) |
| Drug  Carboplatin  Cisplatin | 26 (46.4)  30 (53.6) |
| Adjuvant radiotherapy     Yes     No  Unknown | 7 (12.5)  11 (19.6)  38 (67.9) |
| Exitus     Yes     No | 16 (28.6)  38 (71.4) |

Continuous variables are expressed as median [interquartile range] and categorical variables are expressed as the number of cases (percentage).

**Table S6:** clinicopathological characteristics of the platinum-treated lung adenocarcinoma TCGA cohort **.**

**Figure S1. MAP17 upregulation occurs during lung tumorogenesis and** **is preferentially detected in lung adenocarcinomas.** (A-D) MAP17 mRNA expression in non-tumor and NSCLC samples of different histologic subtypes from different publicly available databases accessible at Oncomine (https://powertools.oncomine.com). NT lung = Lung non-tumoral tissue, LCLC = Large cell carcinoma. (E) MAP17 mRNA expression in lung epithelial immortalized non-tumoral (normal), adenocarcinoma (ADC) and squamous cell carcinoma (SCC) cell lines.

**
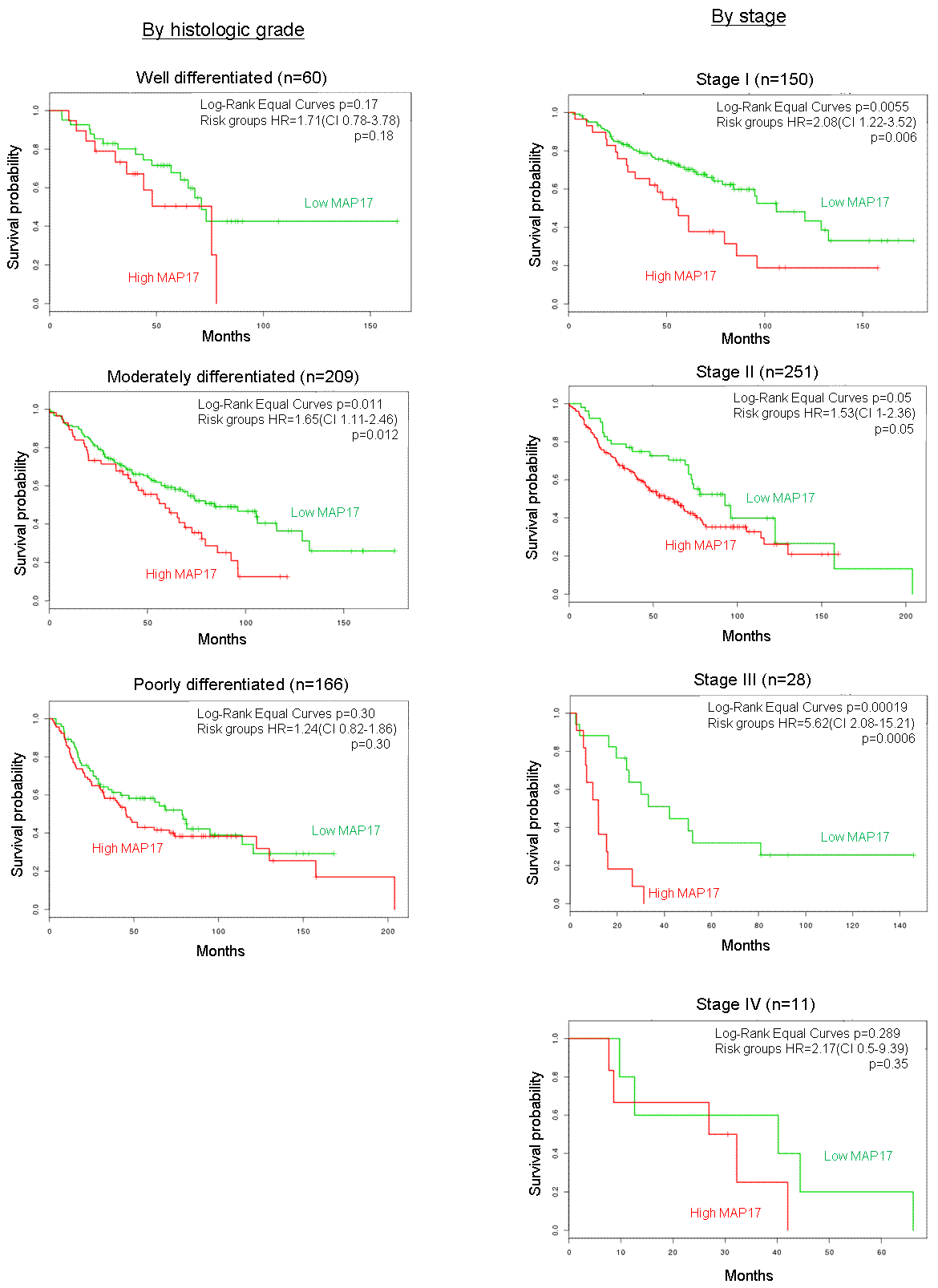
**

**Figure S2.** Analysis of the survival probability according to MAP17 expression in differeng grades or stage of Lung cancer tumors in the Lung Metabase database (n=1053).

**Figure S3:** Relationship between MAP17 mRNA levels and EGFR mutations (based on supplementary table 5).
